# Timing matters: Distinct effects of nitrogen and phosphorus fertilizer application timing on root system architecture responses

**DOI:** 10.1002/pei3.10057

**Published:** 2021-07-22

**Authors:** Richard van Duijnen, Hannah Uther, Werner Härdtle, Vicky M. Temperton, Amit Kumar

**Affiliations:** ^1^ Institute of Ecology Faculty of Sustainability Leuphana University Lüneburg Lüneburg Germany

**Keywords:** barley, fertilizer, functional traits, rhizobox, root system architecture, root traits, timing

## Abstract

**Aims:**

Although different plant foraging responses to the two macronutrients nitrogen (N) and phosphorus (P) are well researched, the effect of timing of fertilizer application on root system architecture (RSA) remains largely unknown. We, therefore, aimed to understand how RSA of *Hordeum vulgare* L. responds to timing of N and P application.

**Methods:**

Plants were grown in rhizoboxes for 38 days in nutrient‐poor soil and watered with nutrient solution, lacking either N or P, with the absent nutrient applied once either 2/3/4 weeks after sowing. Positive controls were continuously receiving N and P and a negative control receiving both N and P only after 3 weeks. We tracked root growth over time, measured plant biomass and nutrient uptake.

**Results:**

Late N application strongly reduced total root biomass and visible root length compared with continuous NP and late P application. Root mass fractions (total root biomass/total plant biomass) remained similar over all treatments, but relative allocation (% of total root biomass) was higher in lower depth with late N application. Shoot P concentrations remained relatively stable, but the plants receiving P later had higher N concentrations.

**Conclusions:**

Late N application had overall more negative effects on early plant growth compared with late P. We propose that future studies under field conditions should try to disentangle the effect of timing from the nutrient availability on RSA responses and hence ultimately plant performance.

## 1 INTRODUCTION

The increase in food production over the last century was coupled with a steep increase in inorganic fertilizer production and use (Tilman et al., 2002). Although yields increased significantly, excessive fertilizer use also led to multiple negative effects on the environment, such as eutrophication of terrestrial and aquatic ecosystems (Bennett et al., 2001; de Vries et al., 2013). Nitrogen (N) and phosphorus (P) are the major macronutrients essential for plant growth and thus make up the bulk of the fertilizer mass. There are differences in terms of the relative importance of these two elements for plant growth, depending on the background soil fertility of these elements in soils. P stocks in European agricultural soils are generally high because of a surplus of P imports and accumulation of P in fixed form (van Dijk et al., 2016). This residual soil P can provide an important steady source of P to crops over multiple years and is often ignored (Sattari et al., 2012). Hence, in many farming settings, P is often not as limiting to plants as N.

Most studies so far have looked at either the timing of application of single N or P fertilizer elements (Efretuei et al., 2016; Finnan et al., 2019; Scharf et al., 2002; Van Es et al., 2004) or specifically at the effects of *spatial* differences in nutrient availability using nutrient patches on plant performance (Drew, 1975; Hodge, 2004; Li et al., 2014; Ma et al., 2013). Recently, Nabel et al. (2018) used both a spatial and temporal approach, adding maize biogas digestate in patches to the prairie plants *Sida hermaphrodita* and found that roots initially avoided the nutrient patch until ammonium had been converted to nitrite and nitrate, after which massive root proliferation into the patch occurred. To our knowledge, no one has yet studied how relative temporal availability of N and P affect plant performance either via aboveground or belowground plant trait plasticity. A multitude of crop root systems have been investigated for their response to N or P deficiency (Liao et al., 2001; Niu et al., 2013; Trachsel et al., 2013). In general, these responses are linked to the spatial availability of the specific nutrient element. For example, P‐deficient plants allocate more resources into top soil foraging where most of the P is located (Lynch & Brown, 2001). Furthermore, morphological and root system architecture (RSA) responses, such as an increase in lateral root length over the amount of laterals, increase in lateral root elongation steeper and angle of laterals, and an increase in root hair length/density are all common plant responses as a mean to increase P uptake when it is limiting (Haling et al., 2013; Linkohr et al., 2002). In contrast to P‐limited plants, N‐limited plants tend to invest more roots in deeper layers because N (especially in the form of NO_3_^−^ ions) is more mobile in soil than P, and they achieve this by changing root growth angles—a root architectural response (Kumar et al., 2020; Trachsel et al., 2013). Consequently, breeding for these specific adaptations in crops could prove advantageous for productivity in N‐ and P‐deficient soils (Lynch, 2011, 2013).

What about the timing, and the relative timing of N and P application, however? Fertilizers are often applied to crops at the time of sowing, although this is likely to be suboptimal regarding nutrient‐use efficiency due to low initial uptake by seedlings, risk of leaching and gaseous losses (de Oliveira et al., 2018; Wuest & Cassman, 1992). Split N application, whereby N fertilizer is applied successively over time to adapt to the ontology and phenology of the crop to enhance uptake and reduce losses, has alleviated the environmental problems significantly (López‐Bellido et al., 2005; Peng et al., 2012). However, the risk remains that crop establishment will be poor and that yield losses occur if plants are nutrient‐deficient early in their growth (Binder et al., 2000; Grant et al., 2001). Besides the nutrients stored in the seed, the root system is responsible for providing N and P to the whole plant shortly after germination. Understanding how young roots respond to N and P availability relative to one another could improve nutrient use efficiency and sustainability in cropping systems.

Roots are known for their phenotypic plasticity in response to nutrient deficiencies and heterogeneous nutrient supplies (Hodge, 2004). Because nutrients are taken up by plants directly from the soil, nutrient‐deficient plants often invest relatively more resources belowground for nutrient acquisition via altered RSA and interactions with the rhizosphere microbiome. A frequently measured response to nutrient deficiencies, as predicted by resource optimization hypothesis (Bloom et al., 1985), is an increase in the belowground carbon (C) allocation to roots and rhizosphere in relation to the total biomass (Agren, 2003; Hermans et al., 2006). These adaptations are species‐specific and range widely from physiological responses (changes in quality and quantity of root exudates) and morphological responses (root diameter, root hair length, and density) to overall RSA responses (spatial distribution of roots, rooting depth, root orders and length, see Koevoets et al., 2016). RSA responses to nutrient deficiencies have been especially well quantified in the model plant *Arabidopsis thaliana* (Gruber et al., 2013). Heterogeneous nutrient conditions often evoke a root proliferation response in the concentrated nutrient patch (Drew, 1975; Hodge, 2004; Li et al., 2014). In this way, the root system can adapt to environmental conditions, and this proliferation response might also confer a competitive advantage (Robinson et al., 1999).

RSA responses to nutrient deficiencies in model plants are mostly measured in nondynamic experimental setups, however, that do not allow tracking of responses over time (but see Nabel et al., 2018) often with one‐time fertilizer application mixed in the starting soil, continuous application using a solution or in a solid growth medium (Hanlon et al., 2018; Xu et al., 2016). Hydroponics approaches provide an easy method to control the abiotic root environment by replacing the growing substrate with a different combination of nutrients but fail to mimic soil conditions where roots face mechanical impedance and heterogeneity in water and nutrient availability, meaning plants have to place their roots selectively. Very few studies explicitly tested timing of N and P fertilization on the root system (but see Peng et al., 2012 and de Boer et al., 2016), despite the common knowledge in agriculture of the importance of fertilizer timing and split fertilizer application. In addition, spatially explicit studies (finding root proliferation in and around nutrient patches) far outnumber temporally explicit studies, potentially because of the difficulty of observing roots non‐invasively over time. Given the key role of N and P for plant performance, it remains curious that so little attention has been paid to the effects of the timing of their application in relation to plant ontology and phenology. Because of the dynamics between nutrient mobility in the soil and nutrient uptake and depletion rates, it is not clear how roots would respond over time. Knowledge on interactions between root foraging and nutrient deficiencies over time would provide useful mechanistic information for innovative and more sustainable fertilization practices.

The objective of this study was, therefore, to dynamically quantify root responses of spring barley (*Hordeum vulgare*) to relative timing of N and P fertilization, while also assessing the overall shoot response and nutrient uptake. To do this, we set up a 5.5‐week rhizobox experiment and applied N or P at 2/3/4 weeks after sowing, giving a factorial design with three levels of N applied late, while P was added straight away and continuously, and vice versa (P applied late and N applied straight away). The experiment involved one positive control with both N and P supplied continuously, as well as a negative control with both N and P applied only after 3 weeks. We measured the visible root length (VRL) nondestructively at the transparent front plate of the rhizobox over time, and root biomass allocation by depth, total shoot/root biomass, and C/N/P uptake at harvest at the end of the experiment.

We asked the following questions:

1a) How does the RSA of spring barley respond to N being applied later than P and vice versa?

1b) How does the length of time elapsed between fertilizing with one element and the next affect the RSA?

2) How do biomass allocation and plant nutrient (N, P) uptake respond to late N/P fertilization treatments?

We hypothesized that:

1. Late N or P application will result in an increase in VRL compared with the control up to a point that N/P deficiency limits overall plant growth.
  - This response will be more pronounced the later the missing nutrient is applied (root foraging).
  - Root elongation rate will decrease after the missing nutrient is applied.
2.
  - Plants will increase biomass allocation to the root system when either N/or P fertilization occurs later. These responses will differ between late N and late P, however, with an increased investment of root biomass in deeper layers (N) or to top layers (P).
  - Plants will exhibit a decrease in total shoot nutrient uptake and nutrient concentrations in shoot tissue for the nutrient, which was applied later.

## 2 MATERIALS AND METHODS

### 2.1 Experimental setup

The experiment was conducted in the greenhouse of the Leuphana University Lüneburg (Lüneburg, Germany, 53°14′23.8″N 10°24′45.5″E) from June 12, 2017 to July 19, 2017 for a total of 38 days. The average day and night temperature and relative humidity were 21.2, 17.0℃ and 63% and 78%, respectively. Rhizoboxes (58.0 × 26.6 × 2.0 cm^3^, volume approximately 3 L) were filled with a ~5 kg mixture of sand (dried at 60℃, sieved at 2 mm), a nutrient‐poor, calcium carbonate–rich, loess soil from a lignite mine near Jackerath (Jackerath, Germany, 51°05′04.8″N 6°27′38.4″E; dried at 60℃, sieved through 2 mm) and a peat potting soil (Nullerde, Einheitserde Werkverband e.V., Germany) in the ratio of 8:2:1, respectively. We chose this very low–nutrient soil so as to more easily control the relative nutrient applications with fertilizer as well as for a complementary study with and without mycorrhizae (data not shown). Soil was compacted by dropping the rhizobox twice from a set distance of 30 cm height after each liter of soil was added. The soil mixture resulted in a nutrient‐poor soil with a total N content of 170 mg/kg, total P content of 135 mg/kg, available P (Olsen P) content of 10.47 mg/kg (at least three times less than average values for more mesic soils), and a pH of 6.69 (0.01 M CaCl_2_). At the start of the experiment, rhizoboxes were saturated with deionized water and left to drain for 24 h, and irrigated with Hoagland solution for the rest of the experiment.

We sowed spring barley (*H. vulgare* L. cv. Barke, Saatzucht Breun, Germany) at a rate of one plant per rhizobox. Seeds were pregerminated for 24 h on a wet tissue paper, and seeds with a radicle of 1–2 mm were transplanted at 1.5 cm depth. Rhizoboxes were placed at an angle of 45 degrees to promote root growth along the front plate due to gravity. Five rhizoboxes were placed adjacently in a container to prevent light infiltration into the rhizobox, and the front rhizobox was covered with a black plate. The last rhizobox was covered with a white polystyrene plate to prevent high temperatures during the day. The positions of the rhizoboxes were randomized per week.

We used eight different nutrient treatment levels to test the effect of the timing of application and nutrient identity on RSA, biomass accumulation, and nutrient uptake of spring barley. We applied all nutrients, except N or P, continuously with the total amount of lacking N or P applied at once after 2, 3, or 4 weeks. We included a positive control group (C; continuous N and P application) and a negative control (F; both N and P applied after 3 weeks) for a total of eight treatments with five replicates (*n* = 5) resulting in 40 rhizoboxes (see Table 1). Rhizoboxes were irrigated three times per week with modified Hoagland solutions. 120 ml of Hoagland solution per week was added for the first 4 weeks, which was increased to 180 ml per week for the last 1.5 weeks. Full Hoagland solution (macronutrients: 5 mM KNO_3_, 5 mM Ca(NO_3_)_2_, 1 mM KH_2_PO_4_, 2 mM MgSO_4_, micronutrients: 46.3 µM H_3_BO_3_, 9.2 µM MnCL_2_, 0.77 µM ZnSO_4_, 0.36 µM CuSO_4_, 0.01 µM MoO_3_, 50.12 µM FeNaEDTA, pH set to 5.8–6 with 2 M NaOH) was used for the control group. For treatments lacking N or P, the Hoagland solution was modified to keep the osmotic potential and other continuous nutrients equal to the control group. For late N, 5 mM Ca(NO_3_)_2_ and 5 mM KNO_3_ were replaced with 5 mM CaCl_2_ and 5 mM KSO_4_, respectively. For treatments lacking P, 1 mM KH_2_PO_4_ was withheld, and 5 mM KNO_3_ was increased to 6 mM and 5 mM Ca(NO_3_)_2_ to 4 mM to accommodate for the loss of K. For the negative control (F), both these changes to the Hoagland solution were made. The treatments lacking either N and/or P received the respective total amount that the control group received over the 38 days (151.3 mg N and/or 22.3 mg P) at 15, 22, or 29 days after sowing (DAS). N was applied as 5 ml of 2.16 M KNO_3_ and P as 5 ml of 0.144 M KH_2_PO_4_.

**TABLE 1:** List of treatments related to timing of N/P application and abbreviations used throughout this study

Treatment	N and/or P application
C (positive control)	Continuous N+P
F (negative control)	N+P applied at 3 weeks
P2W	P applied at 2 weeks
P3W	P applied at 3 weeks
P4W	P applied at 4 weeks
N2W	N applied at 2 weeks
N3W	N applied at 3 weeks
N4W	N applied at 4 weeks

Note

The nutrient that is not mentioned in the abbreviation is applied continuously.

### 2.2 Measurements and harvest

A “photobox” was built to quantify root length over time by acquiring images of the front plate of the rhizobox (as described in Delory et al., 2018). The photobox consisted of metallic frame to hold the rhizobox, a camera holder at a set distance of 54 cm, and two LED lights (BB&S Pipeline FREE, 4300K, 60 cm length), incorporated within a lightproof container. The inside of the container was lined with black cloth, and together with the LED lights allowed us to illuminate the rhizobox uniformly without reflections of the transparent front window. Starting 5 days after sowing (DAS), pictures were taken three times per week with a digital camera (Canon EOS 5D Mark III) equipped with a 28‐mm lens (Canon EF 28 mm f/2.8) at a resolution of a 5760 × 3480 pixels in JPEG. Afterward, pictures of each sampling day were converted to 8‐bit grayscale, and visible roots along the transparent front plate were drawn manually using the SmartRoot plugin (Lobet et al., 2011) for ImageJ (Schneider et al., 2012). The corresponding Root System Markup Language (RSML) file produced by SmartRoot was converted to a table using archiDART (Delory et al., 2016), and the VRL per day was calculated and plotted using R (R Core Team, 2017).

All plants were harvested 38 DAS for shoot and root measurements. One control plant was excluded from all analyses because of poor growth and visible signs of disease at harvest. Shoots were cut off at the base of the stem and dried at 80℃. Rhizoboxes were opened carefully, and soil layers were cut at 10‐cm depth intervals. These layers were washed with a hose above a 2‐mm sieve. A comb was used to catch washed roots, and the 2‐mm sieve was checked for washed through roots. Washed roots were stored at −20℃ until fine washing with a brush and tweezers to remove the last soil and organic peat material from the roots. A subset of eight randomly washed samples (1 per treatment) was stained overnight with an excess of 1.7 mM neutral red, extensively rinsed after, cut into smaller pieces to avoid root overlap, and scanned at 400 dpi using an Epson Perfection V800 Photo (Epson). Scanned images were analyzed with WinRHIZO (Regent Instruments), using the automated global threshold, and local adjustments were made manually to prevent over‐ or underestimation of the total root length (TRL; Delory et al., 2017). The TRL obtained with WinRHIZO by adding root length from each soil depth interval were then used to correlate with the respective VRL at the harvest date. Afterward, all roots were dried at 60℃ until constant weight and dry weight per depth layer was measured. Root mass fraction (RMF) was calculated as the ratio of root biomass to the total biomass (root biomass + shoot biomass). Relative root biomass per depth interval was calculated by dividing the root biomass of a certain layer (e.g., 0–10 cm) by the total root biomass and expressed as a percentage (%). Shoot material was ground with a ball mill (MM 400, Retsch) to a fine powder, and total C and N was analyzed with an elemental analyzer (Vario EL, Elementar). For total P analysis, 200‐mg plant sample was digested with 3 ml 37% HCl, 3 ml 65% HNO_3_, and 1 ml 30% H_2_O_2_ in a microwave (MARS Xpress, CEM GmbH,). Digested samples were analyzed with an ICP‐OES (Optima 3300 RL, Perkin Elmer Inc.).

### 2.3 Statistical analysis

We used one‐way analysis of variance (ANOVA) to test for the effect of the treatments (fixed factor, different combinations of N and P timing, total of eight levels) on root and shoot biomass, relative root biomass per depth interval, VRL at the end of the experiment, and nutrient‐related measurements such as N%, P%, C:N and N:P ratios, and total N and P uptake. If the ANOVA results were significant (*p* < 0.05), we followed up with a Newman–Keuls post hoc test to determine the significant differences between the treatments at *α* = 0.05 using the R package agricolae (De Mendiburu, 2014). Residual and QQ plots were used to visually assess the normality and homogeneity of variance. We determined the Pearson correlation coefficient between the VRL and TRL and fitted a regression line. All statistical analyses were performed using R 3.4.2 (R Core Team, 2017). Refer to Table S1 for descriptive statistics.

## 3 RESULTS

### 3.1 Plant biomass

**FIGURE 1:**
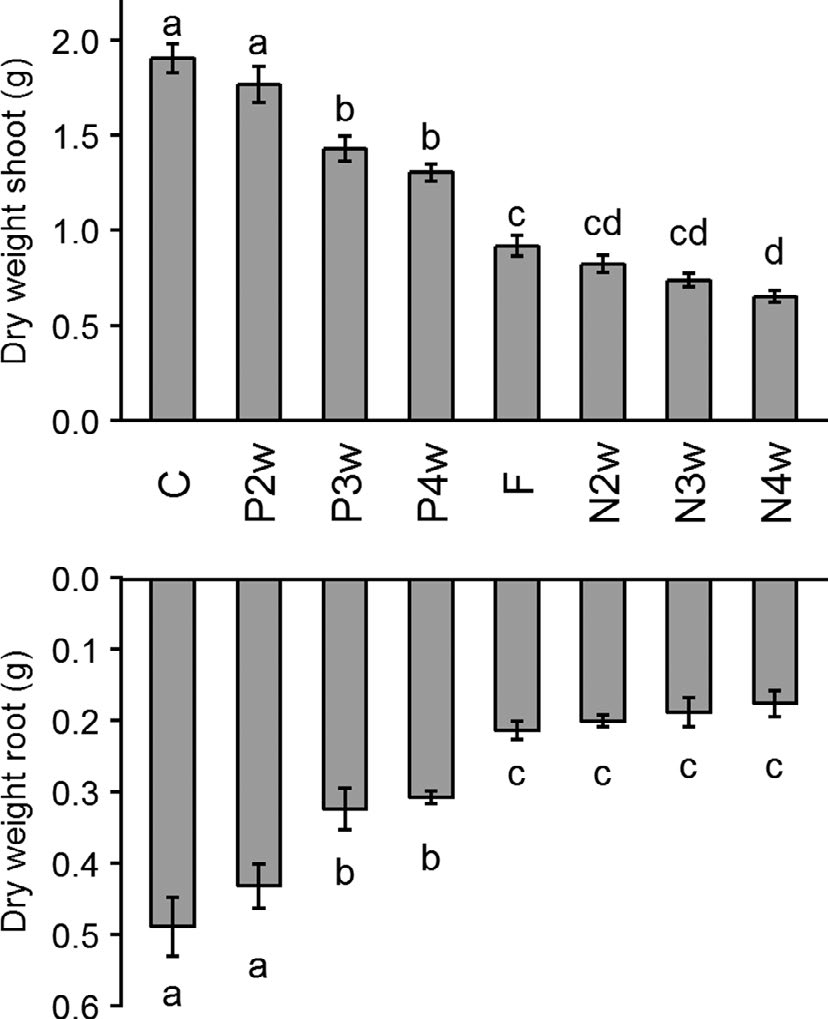
Shoot and root biomass at harvest as affected by the N and P timing treatments; Applying N later than P had a stronger effect on both above‐ and belowground biomass than adding P later than N, but waiting more than two weeks before adding P had a detrimental effect compared to waiting 2 weeks. Treatments are control (C; continuous N and P), P applied after 2/3/4 weeks (P2/3/4W), negative control (F; N and P applied after 3 weeks) and N applied after 2/3/4 weeks (N2/3/4W). Values are mean ± SE (*n* = 4–5). Different letters indicate significant differences between treatments (Newman and Keuls test, *p* < 0.05)

Overall, both for aboveground and belowground biomass, applying N late reduced total biomass on average by 52%–61% compared with control plants supplied with continuous, full Hoagland solution, and how long the plant had to wait for N did not generally matter (Figure 1). Similarly, applying P later also reduced biomass, both for roots and shoots, but only when the time until fertilization occurred exceeded 2 weeks (as with applying N later but less strongly—a reduction of on average 25%–37% in biomass). When P was applied after 2 weeks, we found no significant differences in above‐ or belowground biomass compared with the control plants. Late application of both N and P after 3 weeks (negative control; F) was most similar to, and not significantly different from, N applied after 3 weeks (N3W). However, this treatment had significantly higher shoot biomass compared with sole late N application after 4 weeks (N4W). Interestingly, relative root biomass investment at harvest (expressed by RMF) was not affected by treatments (average RMF by treatment: 0.18–0.21; one‐way ANOVA, *p* = 0.78). Despite similar RMFs, plants in late N treatments invested relatively less into root biomass in the top 10 cm of the rhizobox and more in deeper layers compared with plants of late P and control treatments (Table 2).

**TABLE 2:** Relative root biomass allocation (%) at harvest per depth layer

Depth (cm)	Relative root biomass allocation (%)								ANOVA *p*‐value
	C	P2w	P3w	P4w	F	N2w	N3w	N4w	
0–10	53.3 ± 1.1^a^	52.8 ± 3.4 ^a^	53.6 ± 1.2 ^a^	48.1 ± 2.0 ^ab^	38.5 ± 2.5 ^b^	38.6 ± 5 ^b^	41.7 ± 2.8 ^ab^	37.1 ± 3.3 ^b^	**<0.001**
10–20	13.7 ± 1 ^a^	14.5 ± 0.7 ^a^	14.1 ± 0.2 ^a^	15.5 ± 0.9 ^a^	20.3 ± 0.7 ^b^	20.3 ± 1.7 ^b^	18.21 ± 1.2 ^ab^	18.01 ± 0.5 ^ab^	**<0.0001**
20–30	12.9 ± 0.7	13.0 ± 0.6	11.6 ± 0.8	12.2 ± 0.2	16.4 ± 4.1	15.5 ± 1.1	13.6 ± 2.5	12.2 ± 0.2	0.5406
30–40	6.5 ± 0.6 ^a^	6.9 ± 0.6 ^a^	6.6 ± 0.6 ^a^	8.4 ± 0.5 ^ab^	9.1 ± 1.3 ^ab^	11.5 ± 1.2 ^b^	8.5 ± 1.7 ^ab^	9.4 ± 0.6 ^ab^	**0.0225**
40–50	6.3 ± 0.3 ^a^	5.8 ± 0.8 ^a^	7.4 ± 0.3 ^a^	9.0 ± 0.9 ^a^	9.3 ± 1.2 ^a^	9.9 ± 1.1 ^a^	10.3 ± 1.0 ^ab^	14.9 ± 1.8 ^b^	**<0.0001**
50–60	7.2 ± 1.0	7.1 ± 1.0	6.7 ± 0.8	6.8 ± 0.8	6.4 ± 1.0	4.1 ± 0.5	7.8 ± 3.5	8.3 ± 0.9	0.7035

Note

Values are means ± SE. Different letters indicate significant differences between treatments within the respective layers (Newman–Keuls test, *p* < 0.05).

Bold values indicate a significant result of the ANOVA based on statistical significance at the *p* < 0.05 level.

### 3.2 Visible root length over time

**FIGURE 2:**
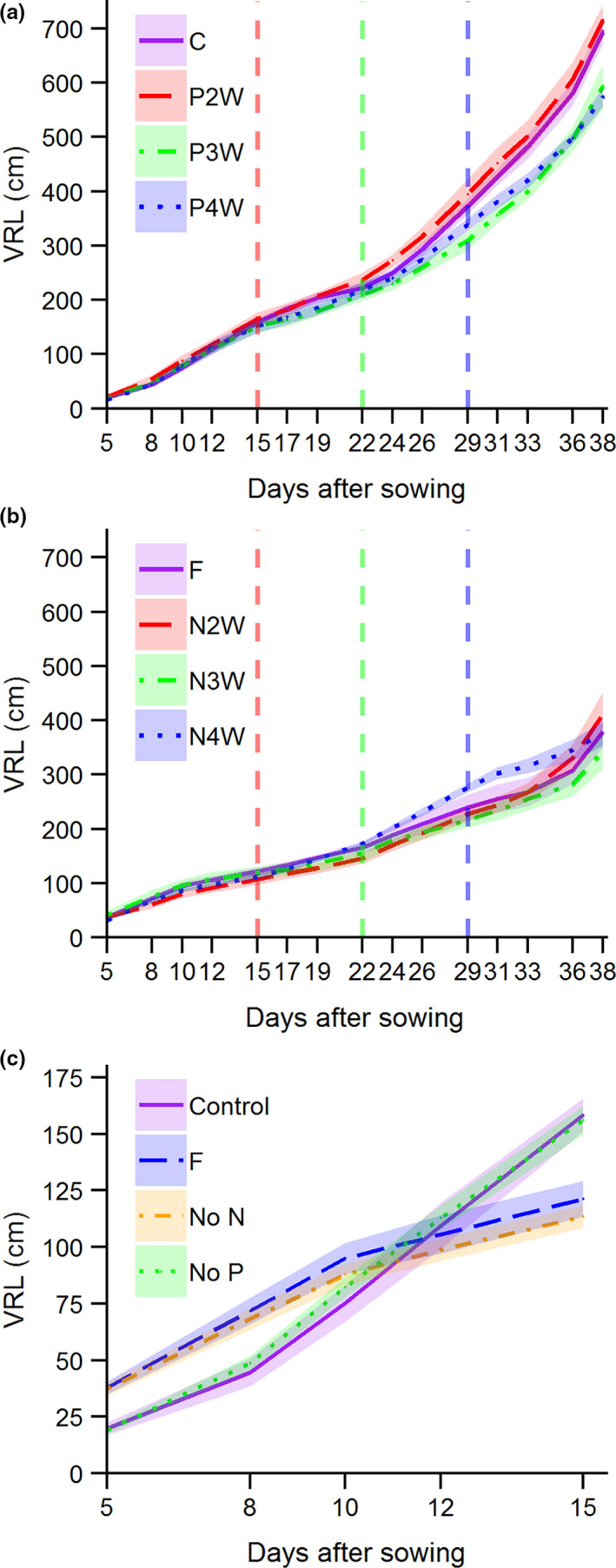
Visible root length (VRL) over time for (a) control (C; continuous N and P) and P applied after 2/3/4 weeks (P2/3/4W) treatments, (b) negative control (F; N and P applied after 3 weeks) and N applied after 2/3/4 weeks (N2/3/4W) treatments, and (c) early response of root length until first N/P application at 15 days after sowing of the late N/P treatments. Vertical lines in panels (a) and (b) indicate the 2, 3 and 4 week time points at which N or P was applied. In panel (c) plants that did not receive N or P yet until 15 days after sowing (treatments N2/3/4W for no N, P2/3/4W for no P, no N and P for negative control) are grouped. Values are means ± SE (*n* = 4–5 for panels a and b, *n* = 4–5 for panel c)

VRL increased similarly over time for late P application compared with the control (Figure 2a), although VRL was slightly reduced from 26 DAS onward when P was applied after 3 or 4 weeks. Late N application showed a different pattern, with an overall much lower VRL (Figure 2b). The VRL increased starting from 22 DAS if N was still not applied after 4 weeks (N4W) compared with N2W and N3W. This was followed by an alignment (lower VRL increase) at harvest date when N was eventually applied after 4 weeks. Figure 2c zooms in on the first 15 days after sowing and shows that initial N‐deficient plants increased their VRL compared with P‐deficient or control plants up to 12 DAS but had lower VRL thereafter. These results show average trends (including errors) over time. We statistically tested the outcome of the different treatments on the VRL at the harvest time only.

### 3.3 Visible root length at harvest and correlation with scanned subsample

**FIGURE 3:**
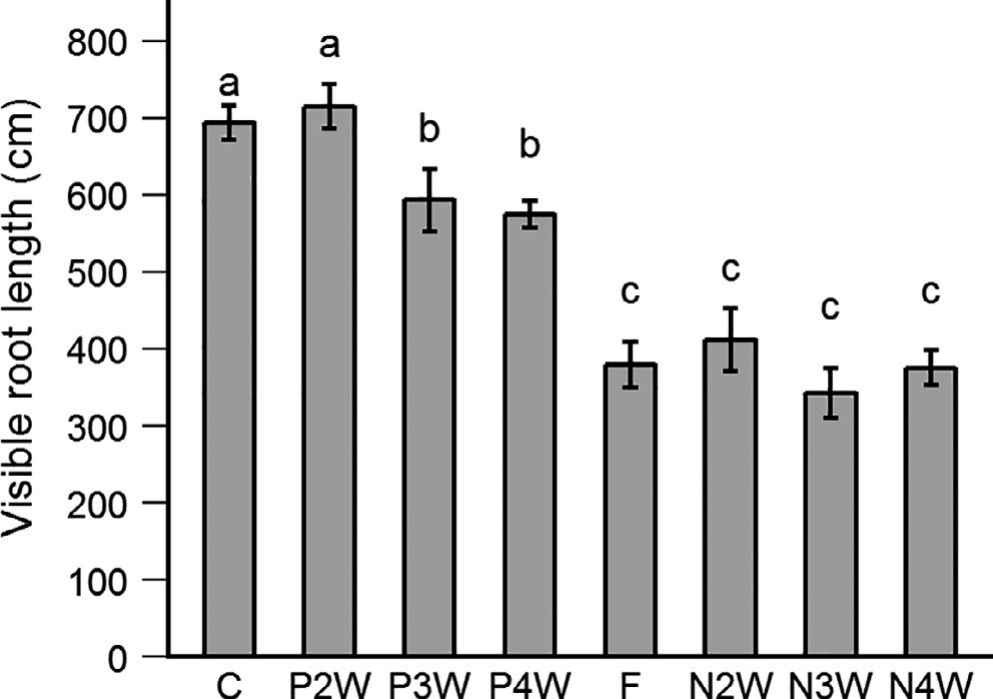
Visible root length at harvest (38 DAS) as affected by the treatments. Adding N late has stronger effect than adding P late. Treatments are control (C; continuous N and P), P applied after 2/3/4 weeks (P2/3/4W), negative control (F; N and P applied after 3 weeks) and N applied after 2/3/4 weeks (N2/3/4W). Values are mean ± SE (*n* = 4–5). Different letters indicate significant differences between treatments (Newman and Keuls test, *p* < 0.05)

All plants, except those that received P after 2 weeks (P2W), had significantly reduced VRL compared with the control (15%–17% reduction for P3W/P4W, up to 41%–51% reduction for late N treatments; Figure 3). Furthermore, late N application had a stronger effect compared with late P application. Although there was a timing effect regarding P application, late N and late N and P had similar VRLs, regardless of the week when N was applied. These patterns are similar to those found in the root biomass at harvest (Figure 1).

**FIGURE 4:**
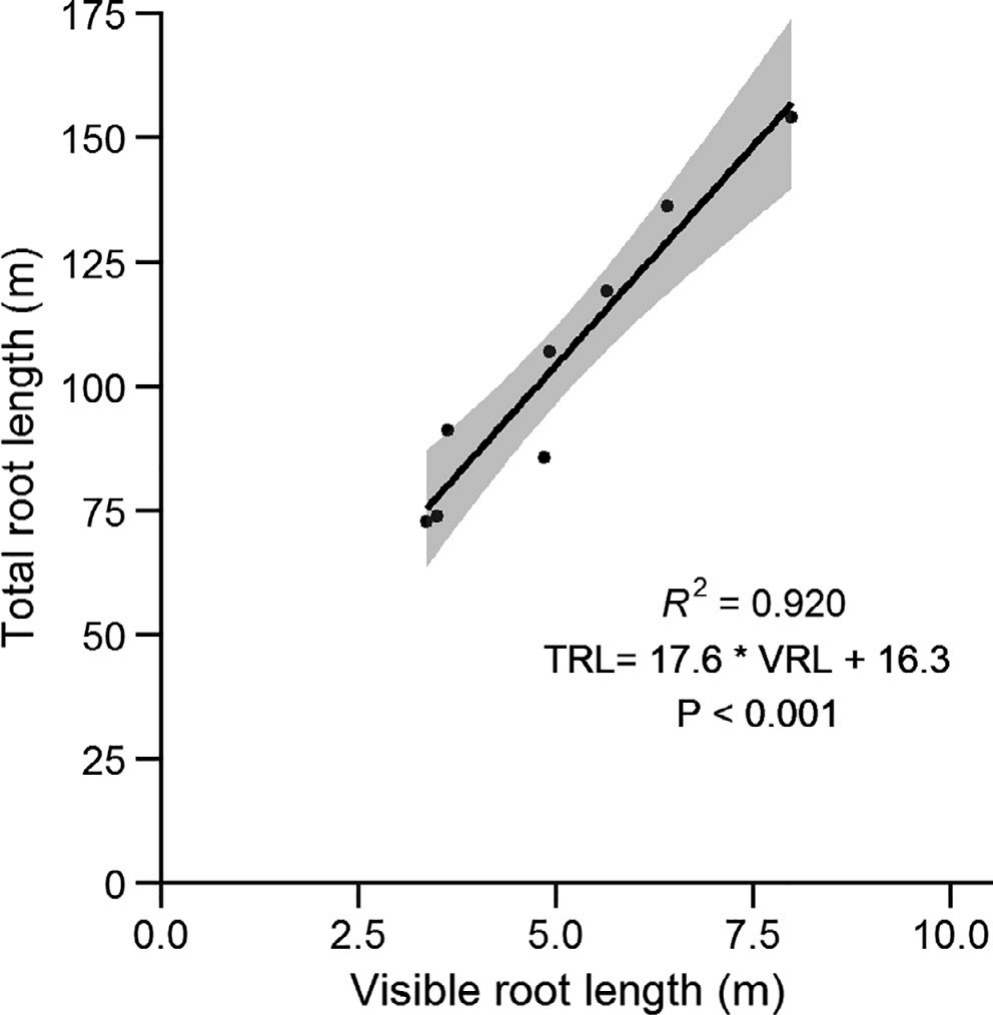
Correlation between visible root length and total root length of randomly selected (1 per treatment) scanned samples measured with WinRHIZO at harvest. The solid line indicates a regression line between visible root length and total root length and the grey shade the 95% confidence interval of the regression fit

The VRL at harvest time correlated well with a subsample of roots scanned with WinRHIZO (Figure 2; *ρ* = 0.959, *p* < 0.001). Overall, 4.5%–5.1% of the TRL (Figure 4, calculated from fitted regression line: TRL = 17.6*VRL + 16.3) was visible along the Plexiglas side of the rhizobox at the end of the experiment.

### 3.4 Nitrogen and phosphorus uptake

**FIGURE 5:**
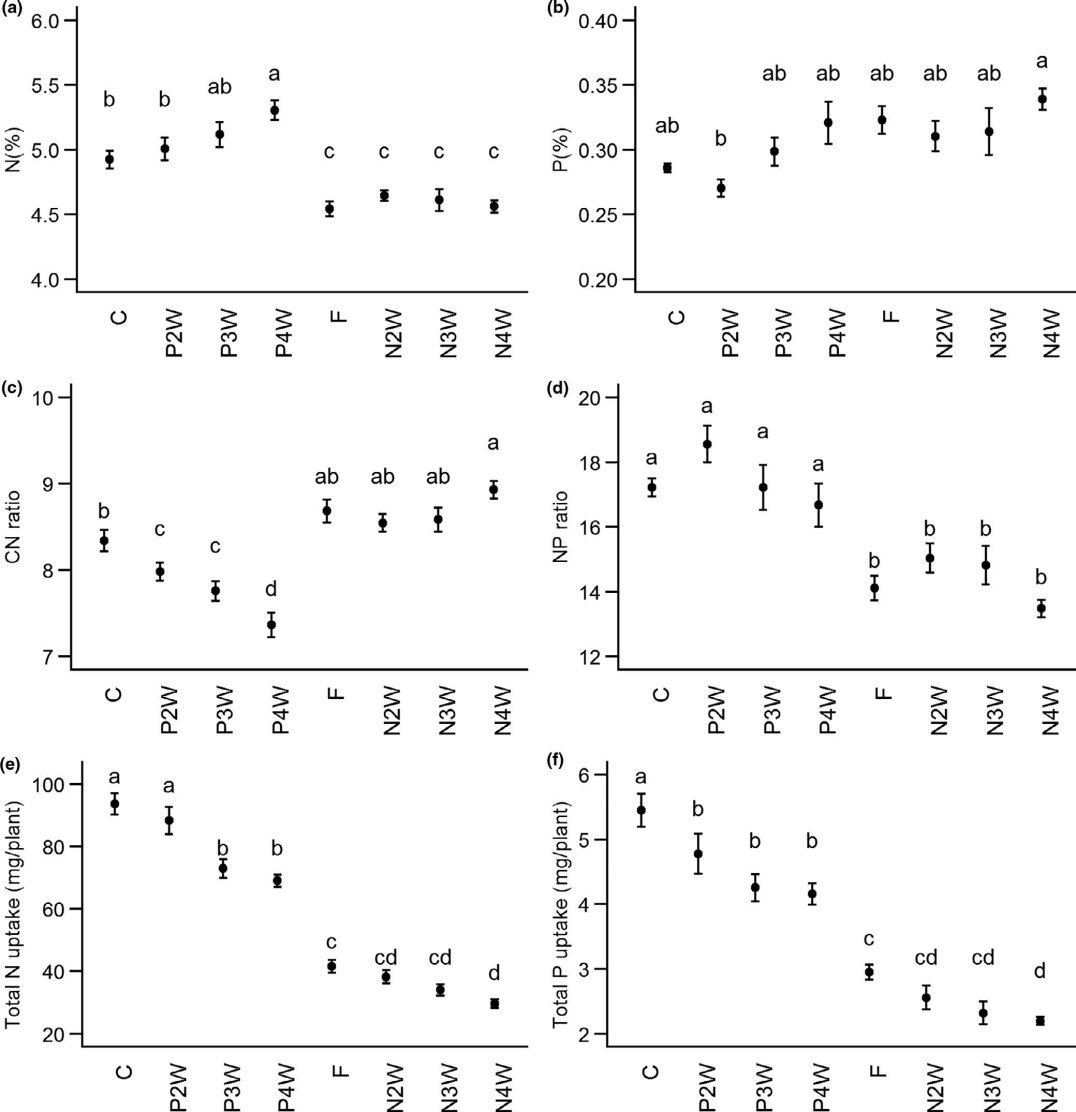
Effect of the treatments on shoot (a) N%, (b) P%, (c) C:N ratio, (d) N:P ratio, (e) shoot total N uptake and (f) total P uptake. Treatments are control (C; continuous N and P), P applied after 2/3/4 weeks (P2/3/4W), negative control (F; N and P applied after 3 weeks) and N applied after 2/3/4 weeks (N2/3/4W). Values are means ± SE (*n* = 4–5). Different letters indicate significant differences between treatments (Newman‐Keuls test, *p* < 0.05)

Late P or N application resulted in two very different responses regarding shoot nutrient concentrations. Late P had a trend of increased N concentrations and corresponding decrease in C:N ratios with increased delay in P application (Figure 5a,c). In contrast, plants within the late N treatments showed no significant differences in leaf N concentrations (%) and C:N ratios irrespective of how much time elapsed before N was applied (Figure 5a,c). P concentrations were less variable across treatments, and only the most contrasting treatments showed significant differences (Figure 5b): Earliest P application (P2W) had the lowest leaf P concentrations, whereas the latest N application (N4W) had the highest. N:P ratios indicate that P late treatments were quite similar to the control, but a trend that P2W was more P‐limited (N:P ratio >18; see Koerselman & Meuleman, 1996), and all N late and the negative control treatments were N‐limited (N:P ratio <15; Figure 5d). Total N and P uptake was severely lower for all late N treatments, mainly due to a decrease in biomass (Figure 5e,f, also see Figure 1). A similar pattern as plant biomass was found for total N uptake in late P, although total P uptake was similar for all three late P treatments (Figure 5e,f).

## 4 DISCUSSION

In this study, we investigated the below‐ and aboveground responses of *H. vulgare* to timing of N and P fertilization. Late P fertilization, beyond 2 weeks after sowing, resulted in a significant decrease in root length and biomass, whereas late N application had a detrimental effect irrespective of time and differences between timing of fertilization mattered less. To our knowledge, this is the first study to explicitly test timing of fertilizer application (including both N and P) on root length over time with dynamic, noninvasive root measurements. The use of rhizoboxes allowed us to see an initial root response to N deficiency in the first 10 DAS and an additional root foraging response when N had still not been applied after 3 weeks.

### 4.1 Fertilizer timing effect on visible root length

We hypothesized that VRL would, by the end of the experiment, increase in all treatments compared with the control treatment because the lack of nutrients would incite root foraging behavior. Although we could not statistically test differences in VRL at different time points (we found no robust method), we identified trends in the curves over time that we discuss here more generally. We expected increased root foraging to occur in the timeframe that the lacking nutrient was not yet applied; after the nutrient was applied there would be a temporary excess of that nutrient thus less need to invest in root exploration. Based on our observations, we did not find any fast root responses, that is, increase or decrease in root elongation within treatments days after N or P was applied, compared with treatments that would have already received this nutrient (Figure 2a,b). However, the first hypothesis seemed from Figure 2c to only be confirmed for the initial elongation response in, mostly, the seminal root system in early N‐deficient plants (until 10 DAS) compared with the positive control treatment. The fact that this response was not observed in the early P‐limited plants could be due to sufficient P reserves in the seeds complemented by soil P. Nadeem et al. (2011) found that maize plants rely solely on seed P up to 5 DAS, a mixture of seed and soil P up to 15 DAS and exclusively on soil P thereafter. This roughly fits our timeline of a decrease in plant performance when P was applied after 3 or 4 weeks. Still, in all late N/P applications, except for P2W, VRL was eventually lower than the control group (Figure 3). Thus, the absolute VRL did not increase significantly due to nutrient deficiencies.

Despite an initial rather strong root response to low N (steeper slopes of the lines over first 10 DAS), we observed another increase in VRL starting around 26 DAS, if N was still a limiting factor at this point, for example, in the N4W treatment (Figure 3b). This response was noticeably absent in plants that had already received N by then. Interestingly, in line with our hypothesis this root elongation response was followed by a decrease in root elongation rate after fertilization after 4 weeks (31 DAS, and these effects lasted until the end of the experiment). These root dynamics, with an increase followed by a decrease, resulted in net similar VRL values for all late N treatments by the end of the experiment (Figure 3). This dynamic would not have been observed if the root length was only quantified at harvest time. We are not aware of any other studies finding such a response to N deficiency and subsequent fertilization. In a slightly similar experiment, in which *Zea mays* plants were initially N‐deficient and thereafter subjected to a split root low/high N treatment, in ‘t Zandt et al. (2015) found that plants increased their root elongation rates in the high N side within 4 days, but these stayed high until the harvest 12 days after N application.

In general, experimental studies on timing of fertilizer application are rare, even more so those that focus on effects on the root system (de Boer et al., 2016; Peng et al., 2012). Peng et al. (2012) found similar results regarding early N‐deficiency in field‐grown maize, namely an initial increase in the root length but severe growth limitation thereafter. De Boer et al. (2016) hypothesized that a delay of N fertilization after a harvest event on a *Lolium perenne* grassland could increase root biomass and N uptake but found no effect for a delay up to 12 days and instead found that fertilization timing had a bigger impact on N dynamics in relation to heavy rainfall events and N leaching. Although this study was done in a slightly different agricultural setup, it shows that the timeframe of response of roots to fertilizer events is an important aspect for improving nutrient use efficiency. The fact that studies that focus on root traits on a temporal scale are rare is most likely due to difficulties of noninvasively quantifying root traits, although nowadays there is a multitude of options available, such as rhizoboxes, mini‐rhizotrons, X‐ray CT, and MRI and PET scans (Atkinson et al., 2019). These methods remain labor‐intensive, although recent advances in machine learning look promising for reducing the labor required. Despite the timing of fertilizer application being more common in agricultural studies (Limaux et al., 1999; Recous & Machet, 1999; Scharf et al., 2002), the focus in many studies is often on aboveground traits, final grain yield, or nutrient budgets and nutrient uptake. For example, Scharf et al. (2002) found no yield decline in maize if N fertilization was delayed until growth stage V11 (11th leaf collar visible) and only a small yield decrease at V12‐16 (12th–16th leaf collars visible). However, this is not directly comparable with our experimental setup, as we chose very low initial nutrient conditions to amplify visible effects of fertilization on root growth. Thus, these conditions did not directly mimic agricultural fields, in which generally higher stocks of soil N are available, and care needs to be taken with extrapolating our results into to the field.

### 4.2 Biomass allocation

As hypothesized, biomass decreased in all treatments compared with the control as expected, but this effect was stronger for delayed N than for delayed P application (Figure 1). This could be because seedlings have a higher N requirement (compared with P) for early growth. Nonetheless, this cannot explain the marginal difference between N fertilization at 2, 3, or 4 weeks. This key result underlines that plants require N very soon after germination and that a delay of 2 weeks was not different in its effect from that of 4 weeks. It could be that plant recovery was faster the more N‐deficient the plants were, as supported by similar N concentrations in the leaf tissue and overall shoot biomass across all N‐late treatment levels, which again could be due to more efficient N uptake and utilization. A number of studies have found that plants are able to maintain similar metabolic and photosynthetic rates under very different N availabilities, and this is often linked to an increase in nutrient use efficiencies (Geiger et al., 1999; Temperton et al., 2003).

Furthermore, RMFs were similar across all treatments, indicating no increased biomass allocation to roots. This was contrary to what we expected, because an often observed response to nutrient deficiency is an increase in biomass allocation to the root system (Hermans et al., 2006; Poorter et al., 2012). According to the resource optimization hypothesis, plants will adapt to the most limiting resources by optimizing their traits to increase uptake of this resource, hence often adjusting their root to shoot ratio under varying environmental conditions. Weiner (2004) states that allocation is better understood as a function of size than time (an allometric perspective). It could be that in our timeframe, RMF was relatively non‐plastic. However, modular plasticity, that is, local adaptation of plant organs as Weiner (2004) defines it, was observed in late N treatment plants, which invested relatively less root biomass in the top 10 cm layer, and more into deeper layers (Figure 1). This supported our second hypothesis that root responses are different for N and P, such that we found evidence for resource optimization for spatial root foraging. Moreover, plants that received N after 4 weeks had significantly increased root biomass at a depth of 40–50 cm, which might also be the extra root production seen in the VRL increase due to N‐deficiency after 30 DAS (as seen in Figure 2b). Because N, applied as nitrate in our experimental setup, is more mobile in the soil, a significant amount of N will most likely have leached to deeper layers of the rhizobox, due to irrigation from the top. Thus, plants would benefit from investing root biomass in deeper layers as well. However, we did not see a similar response for root investment in top layers, in which most of the fertilizer P would accumulate, for late P compared with the control. This could be either due to less stressed plants and a less pronounced P‐deficiency root response, or adaptive responses in other root traits such as root hairs. Root hairs are known to be essential for acquiring P because P is highly immobile in the soil due to adsorption and precipitation, and uptake by bulk flow and diffusion is very low (Gahoonia et al., 1997; Haling et al., 2013). By altering N and P availability in a stoichiometric manner, Kumar et al. (2020) showed that barley had greater proportion of root biomass in deeper soil layers when N was the limiting nutrient and the reverse root response was true for P limitation. In our study, we found no effect of N and P fertilization timing on RMF. As RMF provides information only on resource allocation to root development components, it does not provide information about C investment in root exudation and respiration. Further investigations are needed to account for all the C costs belowground to better understand the root strategies during nutrient uptake over time.

Another plausible reason why we did not find changes in RMF could be the use of crops, in our case *H. vulgare* cv. Barke released in 1996, which have been bred for optimal yields in high‐nutrient‐input agriculture. Decades of selection in wheat (*Triticum aestivum* L.) indirectly selected for smaller, but more N uptake‐efficient root systems (Aziz et al., 2017). Moreover, crops could also have indirectly been selected for less plasticity in the root systems due to breeding under homogeneous, high‐nutrient conditions (Grossman & Rice, 2012).

### 4.3 Nutrient uptake

Total shoot nutrient uptake followed similar trends to the VRL and biomass. However, leaf nutrient concentrations showed two main different patterns. First, we see an increase in N concentrations, and decrease in C/N ratios, the later the P (P2W compared with P3W/P4W) is applied (Figure 5a). However, biomass production was also reduced when P was applied after 3 or 4 weeks (Figure 1). This together points toward more severe P‐limitation as P application was delayed (as expected) but also improved P‐utilization efficiency because total P‐uptake was not significantly different at harvest (Figure 5f). Hence, N accumulated in the leaves, but this did not translate into more growth depending on the severity of the initial P‐deficiency (i.e., time passed until P‐fertilization). Second, all late N treatments had lower N concentrations than the control group due to a lack of initial N availability. Interestingly, the timing of 2, 3, or 4 weeks, N application had no significant effect on N/P concentrations or total N/P uptake (Figure 5a,b,e,f). It could be the plants in these treatments were, thus, severely deficient in N that P uptake was also hindered. Although P concentrations were not significantly higher than the control, N:P ratios clearly indicated less N uptake relative to P (Figure 5d). Concluding, if N is applied in sufficient amounts, P availability matters for total N uptake and plant growth, but if N is not applied in sufficient amounts, P availability does not matter for either total N uptake, total P uptake or plant growth. This means that N is more important than P (at least in early plant development): If plants have no N, P uptake does not improve the plant performance.

### 4.4 Relevance for agronomic practice

The early root system responses in crops are important factors for proper crop establishment. Obviously, in an agricultural context, prevention of yield loss is the most important reason for not delaying fertilizer application, but in an environmental quality context later application is mostly beneficial (Beillouin et al., 2018; Fageria & Baligar, 2005). We showed that initial N deficiency actually increased root length up to a certain point, after which the overall biomass decreased. Certainly, this threshold should be avoided at all cost, and a more complete picture would aid in this. For example, biotic interactions, such as intraspecific competition, can increase root investments (Hodge, 2009) and plant–microbe interactions can alter RSA responses (Vacheron et al., 2013). On the other hand, abiotic conditions, such as water scarcity and drought, can interact with N deficiency and lead to root investments in deeper layers as both resources are commonly found there (Lynch, 2013; Wasson et al., 2012). Lastly, banding of fertilizer is a practical example to make clever use of this early root proliferation response and has been shown to improve nutrient use efficiency, uptake, and yield (Ma et al., 2013). Ma et al. (2013) found root proliferation in localized fertilizer application areas, which lead to an increase in nutrient use efficiency and eventually yield compared with broadcast fertilizer application. It should be noted that the present results are obtained from a greenhouse experiment using rhizoboxes, and we emphasize the need for complementary field level investigations to understand the effects of the timing of fertilizer applications on root responses and overall plant performance. Based on the fact that most farmers are fully aware of the importance of adding nitrogen early whereas phosphorus timing is less critical; however, we suspect that our findings would hold true under field settings too. We require future research especially under field settings to identify whether plants can recover from early nutrient deficiency in later growth stage and what role do rhizosphere microbes play in plant recovery.

## 5 CONCLUSIONS

Timing of fertilizer application significantly affected plant performance, and this was more the case when N was applied later than for P. If plants had sufficient N then P availability mattered for overall plant N uptake, but when plants lacked N then P availability did not matter. Thus, early N availability plays a more important role than early P availability. Late P application did decrease root length and overall plant biomass, but without any clear dynamic root responses. N‐deficiency clearly increased overall root length from germination up to 10 DAS but severely limited growth overall afterward. After four weeks, plants that were still N‐deficient increased their root length a second time, but root length decreased again after the missing N was applied. This study emphasizes the advantages of additional more mechanistic details supplied by dynamic root observations studies, when fertilizer timing is of interest, and provides a basic understanding of the timeframe of spring barley root investments under N/P‐deficiency and consequent supply.

## AUTHOR CONTRIBUTION

Rvd and VT conceived the study. HU and RvD performed the rhizobox experiment and measurements. RvD, VT, and WH analyzed the data. RvD and AK led the writing of the manuscript. All authors contributed to the writing and finalizing of the manuscript.

## CONFLICT OF INTEREST

The authors have no conflict of interest.

## Supporting information

Table S1

## Data Availability

All the data are presented in figures and tables.
