# Supplementary material for: Timing matters: Distinct effects of nitrogen and phosphorus fertilizer application timing on root system architecture responses
Source: Plant Environ Interact. 2021 Jul 22;2(4):194–205. doi: 10.1002/pei3.10057 (PMC10168076; doi:10.1002/pei3.10057)
Supplement: Supplementary file 1 — Table S1 [file PEI3-2-194-s001.docx]

Supplementary table 1: ANOVA table showing effect of temporal variation in N and P availability on total plant biomass (), shoot biomass (), root biomass (), root mass fraction (RMF; ), leaf N (%), leaf P (%), leaf C:N and N:P ratio, total N uptake (), total P uptake (), relative root biomass (%). Df: degrees of freedom. Bold values show statistical significant difference (P < 0.05).

| Variables |  | Df | F value | Pr (>F) |
| --- | --- | --- | --- | --- |
| Total plant biomass | Treatment | 7 | 62.349 | **<2.2E-16** |
|  | Residuals | 31 |  |  |
| Shoot biomass | Treatment | 7 | 62.857 | **<2.2E-16** |
|  | Residuals | 31 |  |  |
| Root biomass | Treatment | 7 | 24.34 | **6.40E-11** |
|  | Residuals | 31 |  |  |
| RMF | Treatment | 7 | 0.562 | 0.7806 |
|  | Residuals | 31 |  |  |
| Leaf N | Treatment | 7 | 15.974 | **1.10E-08** |
|  | Residuals | 31 |  |  |
| Leaf P | Treatment | 7 | 3.347 | **0.00896** |
|  | Residuals | 31 |  |  |
| Leaf C:N | Treatment | 7 | 19.471 | **1.03E-09** |
|  | Residuals | 31 |  |  |
| Leaf N:P | Treatment | 7 | 11.65 | **3.67E-07** |
|  | Residuals | 31 |  |  |
| Total N uptake | Treatment | 7 | 91.104 | **<2.2E-16** |
|  | Residuals | 31 |  |  |
| Total P uptake | Treatment | 7 | 38.856 | **1.30E-13** |
|  | Residuals | 31 |  |  |
| Relative root biomass (0-10 cm) | Treatment | 7 | 8.737 | **6.66E-06** |
|  | Residuals | 31 |  |  |
| Relative root biomass (10-20 cm) | Treatment | 7 | 8.606 | **7.69E-06** |
|  | Residuals | 31 |  |  |
| Relative root biomass (20-30 cm) | Treatment | 7 | 1.303 | 0.2817 |
|  | Residuals | 31 |  |  |
| Relative root biomass (30-40 cm) | Treatment | 7 | 3.612 | **0.005855** |
|  | Residuals | 31 |  |  |
| Relative root biomass (40-50 cm) | Treatment | 7 | 10.201 | **1.46E-06** |
|  | Residuals | 31 |  |  |
| Relative root biomass (50-58 cm) | Treatment | 7 | 0.846 | 0.5583 |
|  | Residuals | 31 |  |  |
